# Leading New Frontiers in Vulva Cancer to Build Personalized Therapy

**DOI:** 10.3390/cancers14246027

**Published:** 2022-12-07

**Authors:** Giacomo Corrado, Giorgia Garganese

**Affiliations:** 1 Gynecologic Oncology Unit, Department of Woman, Child and Public Health, Fondazione Policlinico Universitario A. Gemelli, IRCCS, 00168 Rome, Italy; 2 Department of Life Science and Public Health, Catholic University of the Sacred Hearth, 00168 Rome, Italy; 3 Gynecology and Breast Care Center, Mater Olbia Hospital, 07026 Olbia, Italy

Approximately 3 in 1000 women will receive a diagnosis of vulvar cancer at some point in their lives. More than one in three will be diagnosed at an advanced stage, with reduced chances of survival despite treatment. About 60% of them will already be over 65 years old and will face heavy treatments, even if they are older, with comorbidities and frailty [1].

In addressing this rare disease, we are called to engage on many different and challenging fronts, such as:

- Promote awareness and knowledge of this disease among both gynecologists and women to favor better recognition of early signs of the disease or precursor lesions, as early diagnosis can influence prognosis more than any possible combination of treatments;
- Provide dedicated pathways that include comprehensive care in referral centers, offering teams of trained specialists, experts in the use of new technologies, and the availability of necessary equipment;
- Develop a supportive care system to reduce the impact of the side effects inevitably produced by aggressive treatments such as mutilating surgery or radio-chemotherapy on affected women, with extensive impairment of daily quality of life;
- Expand the provision of palliative therapies, given the tendency of this disease to recur with multiple relapses, requiring additional treatment options even after conventional treatments have been exhausted;
- Implement collaborative networks and data sharing among reference centers to accelerate and make research results more robust.

Each of these points represents a clinical, scientific, and organizational challenge that we will see develop in the coming years along the lines that today’s literature is providing.

The authors of this Special Issue come from one of the European centers with the greatest patient flow and experience in vulvar cancer treatment. In this Special Issue, they focused on collecting the results from several international authors presenting personal experiences and literature reviews or metanalyses, drawing the currently cutting-edge proposals on multiple aspects of vulvar neoplasia.

Significant advances have been made in pre-operative work-up: ultrasound has been reported as able to provide high sensitivity and, more importantly, a reliable negative predictive value (NPV) that could be considered the favored driver in evaluating the performance of an imaging method, since failing to recognize a metastasis and missing the surgical removal could significantly impair prognosis (especially in the lymph node sites).

A highly predictive pre-operative assessment supplies a correct clinical stage and addresses patients towards surgical choices, avoiding, on the one hand, overtreatments, because surgery is burdened by a very high rate of morbidity and, conversely, patients are mostly older and fragile. On the other hand, it avoids step-down treatments, because vulvar cancer is a very aggressive disease for which radical surgery provides a good local control.

In this setting, ultrasound should be considered as an accurate tool for the assessment of nodal status in vulvar cancer to allow patients with a low risk of nodal involvement to receive less invasive surgeries (SNB), thus resulting in fewer postoperative complications of unnecessary lymphadenectomy [2]. The greatest progress in treatment has been the sentinel lymph node (SLN) biopsy, which is considered the gold standard in early-stage disease. The SLN biopsy can be performed in unifocal tumors confined to the vulva (less than 4 cm in diameter), stromal invasion greater than 1 mm, and clinically negative lymph nodes. Similarly, replacing lymphadenectomy with SLN mapping has significantly reduced surgical morbidity [3]. In this setting, the GROINSS-V studies have been pivotal in reducing treatment-related morbidity by finding safe alternative treatment options in patients with early-stage vulvar cancer [4].

In perspective, basing on an accurate preoperative work-up, we might soon see a broadening of the selection criteria for conservative inguinal lymph node surgical treatment [5]. Moreover, reconstructive procedures and microsurgery have had an important evolution in recent years, particularly in reducing lower-limb lymphedema risk. For example, the superficial circumflex iliac perforator flap, including the lymphatic vessels of the flank (L-SCIP), has become a safe, quick, and effective technique in achieving reconstruction of the inguinal area after groin dissection for vulvar cancer, with a relevant positive effect over incidence and severity of secondary lower-limb lymphedema [6].

Furthermore, integration with other treatments, such as radio-chemotherapy, brachytherapy, or electrochemotherapy, has improved overall clinical outcomes, allowing for additional opportunities, even in cases unsuitable for upfront surgery. Based on the available evidence, radiotherapy, for example, with or without concurrent chemotherapy has a relevant role in neoadjuvant, adjuvant, or exclusive treatments [7]. Moreover, modern oncology is increasingly characterized by minimally invasive therapeutic proposals [8]. Especially the elderly, but also patients with comorbidities, can benefit from this type of procedure. Electrochemotherapy is part of this and has proven to be a valid option in the management of patients with relapsed vulvar cancer who have already undergone radical surgery and radio-chemotherapy, showing a positive clinical response and an improvement in QoL without serious adverse events [9,10].

In conclusion, in the management of vulvar cancer, the benefits of personalized approaches by multidisciplinary integration, based on tumor board discussion, are also clearly emerging with the need to address cases to dedicated reference centers. Sample collection and data sharing should be encouraged to achieve a specific molecular profile and to design new translational pathways [11].
